# Molecular analysis of stomach contents reveals important grass seeds in the winter diet of Baird's and Grasshopper sparrows, two declining grassland bird species

**DOI:** 10.1371/journal.pone.0189695

**Published:** 2017-12-20

**Authors:** Mieke Titulaer, Alicia Melgoza-Castillo, Arvind O. Panjabi, Alejandro Sanchez-Flores, José Hugo Martínez-Guerrero, Alberto Macías-Duarte, Jesús A. Fernandez

**Affiliations:** 1 Facultad de Zootecnia y Ecología, Universidad Autónoma de Chihuahua, Chihuahua, Mexico; 2 Bird Conservancy of the Rockies, Fort Collins, Colorado, United States of America; 3 Instituto de Biotecnología, Universidad Nacional Autónoma de México, Cuernavaca, Morelos, Mexico; 4 Facultad de Medicina Veterinaria y Zootecnia, Universidad Juárez del Estado de Durango, Durango, Mexico; 5 Universidad Estatal de Sonora, Hermosillo, Sonora, Mexico; University of Saskatchewan, CANADA

## Abstract

We analyzed the diet of Baird’s Sparrow (*Ammodramus bairdii*) and Grasshopper Sparrow (*A*. *savannarum*) in three different sites and sampling periods across the Chihuahuan Desert in northern Mexico. DNA from seeds in regurgitated stomach contents was sequenced using NGS technology and identified with a barcoding approach using the P6 loop of the *trnL* intron as genetic marker. During each sampling period, we collected random soil samples to estimate seed availability in the soil seed bank. Due to the variability and size of the genetic marker, the resolution was limited to a family level resolution for taxonomic classification of seeds, but in several cases a genus level was achieved. Diets contained a high diversity of seeds but were dominated by a limited number of genera/families. Seeds from Panicoideae (from the genera *Panicum*, *Setaria*, *Eriochloa*, *Botriochloa*, and *Hackelochloa*) contributed for the largest part to the diets (53 ± 19%), followed by *Bouteloua* (10 ± 12%). Depending on the site and sampling period, other important seeds in the diets were Eragrostideae, *Pleuraphis*, Asteraceae, *Verbena*, and *Amaranthus*. The most abundant seeds were not always preferred. *Aristida* and *Chloris* were common in the soil seed bank but these seeds were avoided by both bird species. Baird’s and Grasshopper sparrows did not differ in seed preferences. This work highlights the importance of range management practices that favor seed production of Panicoideae and *Bouteloua* grasses to enhance winter habitat use and survival of Baird’s and Grasshopper sparrows in the Chihuahuan Desert.

## Introduction

Most North American grassland birds are migratory, over-wintering in the Chihuahuan Desert grasslands of Mexico. These grassland birds are among the most threatened groups of birds worldwide [1]. Declining availability of winter habitat is a main factor threatening grassland birds [2]. To reverse population declines of these birds through habitat conservation, it is necessary to investigate their mechanism of habitat selection at the regional (highest) and local (lowest) levels, *sensu* [3]. In this regard, habitat suitability and movement patterns are likely related to resource abundance and distribution [4,5]. Granivorous grassland birds feed almost exclusively on seeds during winter [6]. Consequently, grassland bird abundance is positively associated with seed abundance [7–9]. Therefore, habitat quality for wintering grassland birds must be influenced in part by the species composition of the plant community source of the seed food. At present, habitat quality for grassland birds has been mostly described in terms of structural vegetation characteristics such as grass, forb and shrub cover and height in relation to bird abundance [5,10–13]. However, only a limited number of studies have investigated the importance of plant species composition for the winter diets of grassland birds [14–16].

The diet of Chipping Sparrows (*Spizella passerina*) in the southeastern grasslands of Arizona consists mainly of Lehmann lovegrass (*Eragrostis lehmanniana*), amaranth (*Amaranthus retroflexus*) and purselane (*Portulaca* spp.), while they prefer unarmored forb seeds over armored grass seeds [14]. In contrast, sparrows in the Monte Desert of Argentina prefer grass over forb seeds with differences in selectivity between bird species [15,16]. A comparison of the winter diets of five sparrows in southwestern New Mexico showed that dropseed (*Sporobolus* spp.) is preferred by Chipping Sparrow, Brewer’s Sparrow (*Spizella breweri*) and Vesper Sparrow (*Pooecetes gramineus*), and is important in the diet of Savannah (*Passerculus sandwichensis*) and White-crowned sparrows (*Zonotrichia leucophrys*) as well [6]. Other frequently consumed seeds were feather fingergrass (*Chloris virgata*), stinkgrass (*Eragrostis cilianensis*), amaranth (*Amaranthus* spp.) and carpetweed (*Mollugo verticillata*), with differences between bird species depending on body size, and differences among sites depending on seeds availability [6]. In all studies, sparrows expanded their diets towards the end of the winter by including a larger variety of species or less preferred seeds in their diets, as a possible response to the decreased availability of preferred seeds in the soil seed bank [6,14,16].

Optimal foraging theory suggests that animals select food items in such a way as to maximize energy intake over time [17]. In this regard, laboratory studies show that seed size strongly determines seed selection in such a way that birds select seeds that they can handle most efficiently [18,19]. Larger-billed birds are able to handle a wider range of seed sizes [20,21] and this is reflected in the breadth of the diet [6]. Other determinants of seed selection may be energy content [22] or content of fat [23,24], protein [25,26], carbohydrates [27], water [28], or toxicity [27]. Furthermore, seed selection is influenced by seed abundance and the spatial and temporal distribution of seeds in nature [29]. Grassland bird abundance and diversity is higher in sites with more dense and diverse vegetation [5,30], and bird abundance and seed abundance are positively related [4,8,31]. In a situation with high seed abundance birds may be more selective, specializing on a limited number of preferred seeds, whereas in a situation of low seed abundance birds may expand their diet and include less preferred seeds [18]. Rainfall is the most important determinant of variability in seed production between years [32]. Therefore, which seeds are consumed and how selective birds are may vary between years and sites.

Information on diet provides a means to evaluate habitat quality of grasslands across the wintering grounds and provides a tool for habitat management, a key strategy in grassland bird conservation plans. Here we studied the diet of Baird’s Sparrow (*Ammodramus bairdii*) and Grasshopper Sparrow (*Ammodramus savannarum*) under natural conditions in three different time periods and sites across the Chihuahuan Desert. Baird’s and Grasshopper sparrows are two declining sparrow species [33] that frequently co-occur on the wintering grounds [34]. Although IUCN classifies these bird species as Least Concern, both species have witnessed steep declines in their populations since 1966 and grassland birds in general have declined more than any other North American bird guild over the last 4 decades [33]. Both bird species are listed as birds of Conservation Concern by US Fish and Wildlife Service [35], Chihuahuan Desert priority birds by the Rio Grande Joint Venture [36], and Watch List species by Partners in Flight [37]. In addition, Baird’s and Grasshopper sparrows are grassland obligates [38] and may serve as flagship species for other grassland birds such as the Sprague’s Pipit (*Anthus spragueii*) that uses similar habitat and is listed as Vulnerable according to IUCN classification [39]. Both require grasslands in good condition with tall grasses and dense cover [5]. These birds have comparable body morphology but differ in bill size, with Grasshopper Sparrow having a larger bill than Baird’s Sparrow, which could possibly lead to differences in seed selection between the two species [40].

In contrast to previous studies, we used Next-Generation Sequencing technology and a DNA barcoding approach to identify seed species in regurgitated stomach and crop contents. The use of high-throughput sequencing and DNA barcoding to identify diet components is becoming widespread because it is more objective and effective than morphological identification methods, providing fewer misidentifications of similar food items [41,42]. Our objectives were to (1) identify common seed species in the diet of Baird´s and Grasshopper sparrows, and (2) compare seeds in the diet to seeds in the soil seed bank to obtain information on seed selection by these two bird species.

## Materials and methods

### Study sites

Our study took place at three different locations in the Chihuahuan Desert of Mexico: Ecological Reserve “El Uno” (30°51’34” N, 108°27’17” W), the experimental ranch of the Autonomous University of Chihuahua “Teseachi” (28°32’38” N, 107°26’45” W), both in the state of Chihuahua, and a private ranch “Santa Teresa” (26°17’55” N, 10°09’54” W) in the northwestern portion of the state of Durango. El Uno is dominated by *Bouteloua* spp. and *Aristida* spp. (S1 Appendix) and is grazed by bison (*Bison bison*). Teseachi is dominated by *Bouteloua gracilis* (S1 Appendix) and is grazed by cattle. Santa Teresa is dominated by *Bouteloua* spp. and *Pleuraphis mutica* (S1 Appendix) and is grazed by cattle and horses.

### Data collection

#### Stomach and crop contents

We caught Baird’s and Grasshopper sparrows using mist-nets that were placed in (semi-) open grassland areas within the study sites. Sparrows were banded using USGS aluminum bands and we took the following measurements: weight (to the nearest 0.1 g), wing and tail length (mm) using a ruler, molt, age, tarsus (to the nearest 0.1 mm), bill length, width and depth (to the nearest 0.1 mm) using a caliper, and fat (on a visual scale from 0 to 5) [43]. We then induced birds to regurgitate stomach and crop contents by flushing the stomach with warm water following [6]. We released the birds immediately after the sample was taken. Each site was sampled three times: November 2012, January 2013 and January 2014. In January 2014, an additional pasture with Grasshopper Sparrows was sampled within El Uno because there were apparently no Baird’s Sparrows in that site that year but more Grasshopper Sparrows than the previous winter. Initially, we planned to identify the seeds in the regurgitated stomach and crop contents morphologically, following [6] and [14]. Therefore, stomach samples were collected on a coffee filter, dried immediately, and saved in manila envelopes until further analysis in the laboratory. However, we found that we were not able to objectively identify the stomach and crop contents based on morphology only, and decided to use a molecular approach. In November 2012 and January 2013, we took several stomach samples from Savannah Sparrows and Vesper Sparrows that were used to standardize laboratory protocols (see below).

#### Soil seed bank

Although there may be some seeds still available on plants, these sparrows are presumed to consume seeds from the soil seed bank only [6]. We collected random soil seed bank samples of 10 × 5 cm and 0.5 cm depth in each site to estimate seed availability. A minimum of 25 soil samples was collected per sampling location in every sampling period. We separated seeds from soil using a sifter with three levels, and identified and counted seeds under a microscope. We calculated biomass availability for the most common seed species analyzed (see below). Seed mass data were provided by César Méndez-González (unpublished information) or obtained from literature [14,18,29,44].

#### Botanical composition

We characterized the vegetation during the first sampling period using a line-point intercept method with parallel 50 m vegetation transects, dropping a pin every meter and recording all plant species touching the pin [45]. For each study site, we also constructed a reference collection of plants by collecting one specimen of all plant species encountered.

#### Rainfall

Summer precipitation has been correlated to seed production [31] and sparrow abundance [7], and was determined by calculating total rainfall from May to October. Rainfall data were obtained from nearby weather stations of the Instituto Nacional de Investigaciones Forestales, Agricolas y Pecuarias (INIFAP), and the mean of ≥4 surrounding weather stations was calculated to obtain precipitation data for each site.

### Ethics statement

Capture, banding and handling of birds was performed by trained and experienced individuals. The data collection protocol was approved by the Bird Banding Laboratory (BBL; permit number 22415) and the Mexican Secretary of Environment and Natural Resources (Secretaria de Medio Ambiente y Recursos Naturales, SEMARNAT; permit numbers 08788/12 and 09559/13). Birds were released immediately after the stomach and crop sample was taken.

### DNA barcode selection

DNA barcoding has successfully been used in several herbivore diet studies e.g. [46–48] and has been recommended as a more objective way of diet analysis from stomach contents or feces [42]. For plant species, there is not one established barcode, but several regions have been proposed. These include a combination of *matK* and *rbcL*, both in a coding region of the chloroplast DNA [49], the intergenic spacer *trnH*-*psbA* [50], a short chloroplast region called the P6 loop of the *trnL* intron [51], and finally the nuclear regions nrITS and its shorter variant nrITS2 [50]. Of these available barcodes, the g-h region of the P6 loop of the *trnL* intron is a small fragment that has been successfully used in herbivore diet studies and was found to have a good performance with highly degraded DNA [46–48]. Therefore, we chose to use the g-h region of the P6 loop of the *trnL* intron as a barcode in the present study, in which the DNA extracted from the regurgitated stomach and crop samples was of low quality. Additionally, the DNA from stomach and crop samples was contaminated with bird DNA for which a chloroplast barcode was desirable.

### DNA extraction

We extracted DNA from the stomach and crop samples using the DNeasy Plant Mini Kit (Qiagen^®^) following the manufacturer’s protocol. We prepared the samples for DNA extraction under liquid nitrogen using a mortar and pestle. Nitrogen was not poured directly onto the sample. Rather, the mortar was placed inside the nitrogen and the sample was allowed to freeze before further processing. Extraction followed immediately and samples were not allowed to thaw. In some cases where large pieces of seed were still visible after vortexing, we used a micropestle to grind the sample further inside the tube after the buffer had been applied in the first step of the extraction protocol. This sample preparation method resulted in the highest DNA concentrations following several tests with samples from Savannah and Vesper sparrows, using different methods, including a mortar and pestle, a micropestle to grind samples in the microcentrifuge tubes, and direct or indirect nitrogen application. We combined samples to obtain a sufficient amount for extraction by grouping 3–5 samples of the same bird species in one site and sample period (Table 1). The number of samples that was grouped depended on the amount of sample obtained from stomach and crop as well as the total number of samples taken from each bird species within one site and sampling period and therefore varies between groups (bird species × study site × sampling period). Final elutions were performed in 50 μl of buffer AE to obtain a higher concentration. The second elution was performed in a separate microcentrifute tube. We stored the DNA at -20°C until further analysis.

**Table 1:** Collected stomach samples per bird species, study site and sampling period, and number of groups that were formed (between brackets) by combining samples to obtain sufficient material for DNA extraction^a^.

	Baird’s Sparrow			Grasshopper Sparrow		
	Teseachi	El Uno	Santa Teresa	Teseachi	El Uno	Santa Teresa
**Nov 2012**	12 (3)	13 (3)	44 (8)	36 (7)	18 (5)	11 (3)
**Jan 2013**	7 (2)	15 (3)	21(4)	27 (5)	22 (4)	19 (4)
**Jan 2014**	21 (5)	0	13 (3)	16 (4)	33 (5–4)^b^	0 (0)

^a^This may be variable due to the variation in the amount of stomach and crop contents that was obtained

^b^Samples were taken in two different pastures within the ranch: El Uno-Centro (same pasture than other sampling periods) and El Uno-Los Ratones.

### DNA amplification and next-generation sequencing

An amplicon for the g-h region of the P6 loop of the *trnL* intron [51] was obtained for each sample, following the 16S Metagenomic Sequencing Library Preparation Kit (Illumina, California) protocol with modifications to generate the amplicon with sequencing tags and adapters. For each pool of samples, two PCR reactions were performed, the first one amplifies the g-h region of the P6 loop of the *trnL* intron and the second is to attach the Illumina tag to identify each pool of samples and the sequencing adapter needed for the Illumina sequencing protocol. We included negative controls with only the PCR mix and water and positive controls with plant material from the reference collection.

Identification tags were designed for each bird species × study site × sampling period combination. The first amplification using the P6 loop of the *trnL* intron round involved 1 cycle of 10 min at 95°C, 30 cycles of 30 s at 95°C, 30 s at 55°C, 30 s at 72°C and a final cycle of 5 min at 72°C, and was carried out in a final volume of 20 μL using 4 ng of DNA and 1 μL of each primer in addition to DMSO at a final concentration of 3%. The results were verified with an agarose gel at 1%. The product of the first PCR was purified using Agencourt AMPure XP Beads (Agencourt^®^) and resuspended in a volume of 10 μL. After this we pooled the bird species, study site and sampling period samples. To do this the concentration of each PCR product was quantified using a Qubit High Sensitivity Assay (Qubit^®^) and pools were formed by combining an equal volume for every sample at a concentration of 1 nM. The second amplification round was performed in a volume of 25 μL using 5 μL of every pool, 2.5 μL Nextera XT Index Primer 1 and 2.5 μL Nextera XT Index Primer 2 (N7XX and S5XX, respectively; Illumina^®^), 2.5 μL of water and 12.5 μL of 2X Phusion PCR Master Mix (Phusion^®^). The mixture was denatured at 98°C for 30 s followed by 8 cycles of 10 s at 98°C, 15 s at 55°C and 15 s at 72°C and a final cycle of 5 min at 72°C. PCR products were then purified using Agencourt AMPure XP Beads (Agencourt^®^) and resuspended in a volume of 20 μL of eluation buffer. We determined the DNA concentration in every pool using a Qubit High Sensitivity Assay (Qubit^®^) to prepare 4 nM of every pool. After tagging, the final concentration was determined using RT-PCR with the Universal KAPA Library Quantification Kit (KAPA Biosystems^®^) for Illumina platforms. Finally, pools were combined and prepared for sequencing with the Illumina MiSeq (Illumina^®^) using a kit for 150 cycles. Sequence data were deposited in the NCBI database (Accession PRJNA396956).

We amplified and sequenced the same DNA barcode region of 18 reference plant species. The PCR program involved 1 cycle of 10 min at 95°C, 45 cycles of 30 s at 95°C, 30 s at 55°C and 30 s at 72°C and a final cycle of 5 min at 72°C. We sequenced reference plants by the Sanger method [52] because only one sequence per sample needed to be obtained. We selected the 18 reference plants based on their abundance in one or more study sites as detected in vegetation transects (S1 Appendix) or because they have been found to be common in the diet of related sparrow species [6]. These plants were: *Bouteloua gracilis*, *B*. *curtipendula*, *Bothriochloa barbinodis*, *Setaria macrostachya*, *Muhlenbergia rigida*, *Schkuhria pinnata*, *Panicum obtusum*, *Amaranthus palmeri*, *Eragrostis cilianensis*, *Aristida adscencionis*, *Chenopodium album*, *Digitaria californica*, *Pleuraphis mutica*, *Chloris virgata*, *Mollugo verticillata*, *Sporobolus airoides*, *Portulaca pilosa*, and *Lycurus phleoides*. Accession numbers for the nucleotide sequences of the reference plants are MF598356 –MF598373.

### Identification of seeds in stomach and crop contents

We identified DNA sequences by comparison to the sequenced reference collection as well as a customized database constructed by taking the target sequences from GenBank (NCBI) from all plants encountered in either the reference collection of plant species collected in the field, vegetation transects, or soil samples. Including the reference collection, the customized database included 166,834 sequences (DOI 10.17605/OSF.IO/AYJNS). Read alignment against the reference database was performed using the program SMALT 0.7.6. The database was indexed using a kmer size of 11 and a step size of 2 (index option, -k 11 and -s 2). The mapping was performed with the -x parameter to report only the best hit at a minimum of 70% of identity. The generated SAM files were parsed to count the reads mapping to a certain reference in the database. All the reads were evaluated based on their CIGAR score, where all the read bases align to a reference with no mismatches (perfect mapping reads) and were assigned to the species annotated for the reference. Because the genetic barcode did not always map perfectly to the reference, we could not discriminate well between species. Therefore, those reads were assigned at genus level. We calculated the number of reads per genus for each group (bird species × study site × sampling period) as well as the proportion of the total number of reads per genus. In the cases where the resolution of the genetic barcode was insufficient to discriminate reliably between seed species or genera, a family taxonomic classification was used.

### Statistical analysis

We used R 3.3.1 [53] for all statistical analyses. To investigate whether birds are selective in their diet, we analyzed the data using a Dirichlet regression with *seed composition* (proportion of every seed) as the dependent variable and *SAMPLE ORIGIN* (diet or soil) as independent variable. A Dirichlet regression is a type of compositional analysis based on the Dirichlet distribution and does not assume a multivariate normal distribution or homoscedasticity of the data [54]. Like all compositional analyses, Dirichlet regression uses a logarithmic link function of the compositional variable. This transformation overcomes potential problems with non-independence of proportional data [55]. Because of this transformation, it is not possible to have zeros in the data, therefore we replaced them by a small value [55]. In some cases the proportion of reads was smaller than 0.001, therefore zero values were replaced by 0.0001. The compositional response was based on the most common seeds in either diet or soil samples (S2 Appendix). The criteria used to select these seeds was an abundance of 10% or more in at least one group (bird species × study site × sampling period) or soil in one sampling period. The Dirichlet regression tests the significance of the regression coefficients (*B*) for each compositional variable (seed) with a *z*-test. Significantly positive regression coefficients indicate a that the proportion of the seed is larger in the diet than in the soil and significantly negative regression coefficients indicate that the proportion of the seed is larger in the soil than in the diet.

To test the hypothesis that selectivity differs between bird species, we performed a Multivariate Analysis of Variance (MANOVA) on the *log-ratios diet-soil* (difference between the log-transformed proportion in the diet and the log-transformed proportion in the soil) for the selected seeds, with *BIRD SPECIES* as factor. We included *precipitation* as a covariate because we expected that rainfall would influence seed species abundance and diversity. Samples were taken in different study sites and sampling periods. Our main interest here was to effectively capture the variation in diet composition, not necessarily differences between sites and sampling periods. However, the factor study site may encompass several ecological or environmental variables that could influence bird diets such as precipitation and vegetation type. To control for the effects of these unmeasured variables, we added *STUDY SITE* and *SAMPLING PERIOD* as factors to the model. We used Wilk’s λ as test statistic. We obtained the final model through backward deletion of non-significant terms. The assumption of multivariate normality of the residuals was checked graphically (not illustrated).

We analyzed differences in seed availability (seeds ha^-1^) between sites and sampling periods with an Analysis of Variance (ANOVA) with *STUDY SITE* and *SAMPLING PERIOD* as factors. Previous studies have found a decrease in abundance of preferred seeds from early to late winter [6,16,29]. To test whether seed availability differed between November 2012 and January 2013 in our study, we compared seed availability between sampling periods using a Tukey HSD post-hoc test. To test whether summer rainfall was related to seed availability we calculated the Pearson correlation coefficient between *rainfall* (mm) from May to October preceding the data collection and *seed availability* (seeds ha^-1^) in the soil seed bank

## Results

A total of 146 Baird’s Sparrow samples and 182 Grasshopper Sparrow samples were collected, with notable differences in the number of samples between study sites and sampling periods (Table 1). Mist-netting efforts indicated that bird abundance differed between years. The winter of 2013–2014 was milder with more rainfall in the previous summer (Table 2) which in turn was reflected in higher bird abundance that winter. However, Santa Teresa received less summer rainfall preceding the winter of 2013–2014 and the ranch was heavily grazed in 2013, resulting in fewer birds and no Grasshopper Sparrows. In El Uno, overall bird abundance was much higher in January 2014 as compared to the other sampling periods; but intriguingly, there were no Baird’s Sparrows whereas the previous year there were. In Teseachi, grassland condition was good in both winters and both species were found in all three sampling periods. However, Baird’s Sparrows were less abundant during the winter of 2013–2014.

**Table 2:** Precipitation (mm) from May to October preceding the two sampling seasons (winter of 2012–2013 and 2013–2014).

	Santa Teresa	Teseachi	El Uno
**Season 1**	361.75	317.15	194.81
**Season 2**	301.25	472.43	260.33

### Diet samples

The sequencing results indicated a low resolution of the genetic barcode. Sometimes birds would appear to be consuming seeds that were not present at a study site based on the three different characterization methods (soil samples, vegetation transects and reference collection). However, a seed of a related species was usually present at those sites. This was especially the case with seeds of Panicoideae (*Botriochloa* spp., *Eriochloa* spp., *Hackelochloa* spp., *Panicum* spp., and *Setaria* spp.), Eragrostideae (*Eragrostis* spp., *Lycurus* spp., and *Muhlenbergia* spp.) and Asteraceae (*Hypochaeris* spp., and *Machaeranthera* spp.) We therefore analyzed these seeds at the family taxonomic level and other seeds at the genus level. Between 84 and 94% of sequence reads were identified to this level, depending on the pool (Table 3).

**Table 3:** Sequencing yield per sample and percentage of mapped reads to a reference. The reported reads are those that passed an average base quality cutoff value of 30 (q ≥ 30).

Sample (pool)^a^	Number of reads (q ≥ 30)	Mapped reads (%)
**BAIS, STE, Nov 2012**	3534032	3280437 (92.82%)
**GRSP, STE, Nov 2012**	316490	289468 (91.46%)
**BAIS, TES, Nov 2012**	304364	283609 (93.18%)
**GRSP, TES, Nov 2012**	100273	85131 (84.90%)
**BAIS, UNO, Nov 2012**	262910	240577 (91.51%)
**GRSP, UNO, Nov 2012**	223029	199296 (89.36%)
**BAIS, STE, Jan 2013**	389963	365473 (93.72%)
**GRSP, STE, Jan 2013**	421051	392760 (93.28%)
**BAIS, TES, Jan 2013**	406244	382801 (94.23%)
**GRSP, TES, Jan 2013**	428691	398755 (93.02%)
**BAIS, UNO, Jan 2013**	425142	403093 (94.81%)
**GRSP, UNO, Jan 2013**	386686	358330 (92.67%)
**BAIS, STE, Jan 2014**	447858	412532 (92.11%)
**BAIS, TES, Jan 2014**	428304	395656 (92.38%)
**GRSP, TES, Jan 2014**	428372	402987 (94.07%)
**GRSP, UNO-Centro, Jan 2014**	427170	393436 (92.10%)
**GRSP, UNO-Lora, Jan 2014**	354111	330501 (93.33%)

^a^BAIS = Baird’s Sparrow, GRSP = Grasshopper Sparrow, STE = Santa Teresa, TES = Teseachi, UNO = El Uno

The main seeds consumed by Baird’s and Grasshopper sparrows in all sites belonged to Panicoideae, as these seeds represented 10 to 84% of total reads in the diet samples, depending on site, sampling period and bird species (Table 4; S3 Appendix). Panicoideae in the study sites include species from the genera *Panicum*, *Setaria*, *Botriochloa*, *Eriochloa*, and *Hackelochloa*. Soil samples indicate that species from the genus *Panicum* in Santa Teresa, species from the genera *Eriochloa* and *Panicum* in Teseachi and species of *Panicum* and *Setaria* in El Uno were the most common Panicoideae. Other commonly consumed seeds in all sites belonged to the genus *Bouteloua*, ranging from 0 to 35% of total reads (Table 4; S3 Appendix). For other seeds, consumption was more variable between study sites, sampling periods and bird species. Next to Panicoideae and *Bouteloua*, the genus *Pleuraphis* and the family Eragrostideae were common in Santa Teresa and El Uno. Eragrostideae were also frequently consumed in Teseachi, along with seeds of the genus *Verbena* (Table 4; S3 Appendix).

**Table 4:** Most common seeds in diet samples (mean percentage of total sequence reads ± S.D.) from Baird’s (BAIS) and Grasshopper sparrows (GRSP) per site, averaged over the sampling periods (n^a^).

	Santa Teresa		Teseachi		El Uno—Centro		El Uno—Lora
	BAIS n = 3	GRSP n = 2	BAIS n = 3	GRSP n = 3	BAIS n = 2	GRSP n = 3	GRSP n = 1
**Panicoideae**	44.03 (8.03)	22.80 (17.41)	45.00 (3.58)	52.36 (12.95)	78.98 (6.49)	70.49 (13.18)	63.99 (--)
***Bouteloua* spp**.	4.07 (2.60)	33.31 (1.95)	15.21 (13.00)	10.76 (7.94)	3.29 (4.50)	0.28 (0.36)	5.90 (--)
***Pleuraphis* spp**.	16.67 (12.30)	8.15 (1.10)	5.17 (1.16)	3.95 (1.24)	2.65 (3.54)	6.33 (3.52)	3.48 (--)
**Eragrostideae**	14.10 (9.88)	5.04 (3.36)	10.93 (3.85)	7.67 (1.16)	5.25 (6.99)	11.77 (8.10)	4.81 (--)
**Asteraceae**	8.48 (8.93)	8.78 (11.75)	2.44 (4.07)	4.62 (6.66)	1.21 (0.78)	0.71 (0.45)	0.19 (--)
***Verbena* spp**.	0.66 (1.02)	0.05 (0.06)	8.38 (12.98)	4.38 (6.94)	0.02 (0.02)	0.03 (0.04)	0.00 (--)
***Amaranthus* spp**.	0.51 (0.88)	0.00 (0.00)	0.00 (0.00)	0.00 (0.00)	0.01 (0.01)	0.34 (0.58)	14.11 (--)

^a^Diet samples were pooled for molecular analysis (see Methods); n refers to the number of pools.

### Soil seed bank

The soil seed bank contained up to 108 different seed species. On average, seed availability was 1.5 × 10^9^ seeds ha^-1^ for the first season and 6.4 × 10^9^ seeds ha^-1^ for the second season. Seed availability differed between sampling periods (*F* = 11.26, *df* = 2, 10 *p* = 0.006) but not between sites (*F* = 1.45, *df* = 3, 10, *p* = 0.355). *Post-hoc* comparisons show that seed availability did not differ from early to mid-winter during the first season (Tukey HSD, November 2012 *vs*. January 2013: *p* = 0.959) but was higher for the second season (Tukey HSD, November 2012 *vs*. January 2014: *p* = 0.015; January 2013 *vs*. January 2014: *p* = 0.010). Precipitation did not correlate with seed availability (seeds ha^-1^, *r* = -0.121, *p* = 0.739). Common seeds in the soil seed bank of all sites were seeds of the family Panicoideae, and the genera *Aristida* and *Bouteloua* (Table 5, S1 Appendix). Interestingly, *Aristida* seeds were rarely found in the diet samples (Table 6; S3 Appendix). Other seeds differed between sites. In the last sampling period, an additional site was sampled in El Uno with a very high production of *Amaranthus* seeds. Asteraceae were common in Santa Teresa but absent in the soil in Teseachi. Teseachi was the only site containing *Verbena neomexicana*, and *Pleuraphis mutica* was the only species of the genus *Pleuraphis* that was present and it was only present in Santa Teresa and one site at El Uno, “El Uno-Centro" (Table 5, S1 Appendix).

**Table 5:** Mean (± S.D.) biomass (kg/ha) of the most common seeds in the soil seed bank of each study site that were used for analysis averaged over the three sampling periods.

	Santa Teresa	Teseachi	El Uno—Centro	El Uno—Ratones
	n = 3	n = 3	n = 3	n = 1
**Panicoideae**	80.85 (83.03)	353.82 (205.88)	221.59 (297.23)	737.50 (--)
***Bouteloua* spp**.	105.60 (22.54)	69.33 (20.24)	127.05 (132.61)	499.97 (--)
***Pleuraphis* spp**.	29.85 (13.58)	0.00 (0.00)	15.11 (21.74)	0.00 (--)
**Eragrostideae**	11.27 (18.26)	33.86 (22.46)	19.23 (33.30)	137.68 (--)
**Asteraceae**	300.34 (336.78)	0.27 (0.23)	50.35 (7.73)	62.25 (--)
***Verbena* spp**.	0.00 (0.00)	16.42 (13.43)	0.00 (0.00)	0.00 (--)
***Amaranthus* spp**.	0.09 (0.15)	0.00 (0.00)	2.10 (1.05)	1111.68 (--)
***Chloris* spp**.	82.69 (51.16)	49.11 (45.10)	1.76 (1.64)	14.56 (--)

**Table 6:** Comparison of the average proportion (± SD) of the nine most common seeds in diets and soil seed bank based on their total.

	Baird’s Sparrow		Grasshopper Sparrow	
	n = 8		n = 9	
	Diet	Soil	Diet	Soil
**Panicoideae**	0.60 (0.18)	0.27 (0.21)	0.61 (0.23)	0.31 (0.19)
***Bouteloua* spp**.	0.09 (0.10)	0.18 (0.10)	0.15 (0.18)	0.20 (0.08)
***Pleuraphis* spp**.	0.10 (0.11)	0.03 (0.04)	0.07 (0.03)	0.03 (0.04)
**Eragrostideae**	0.12 (0.08)	0.02 (0.03)	0.09 (0.06)	0.03 (0.03)
**Asteraceae**	0.05 (0.07)	0.18 (0.16)	0.05 (0.09)	0.12 (0.12)
***Verbena* spp**.	0.04 (0.09)	0.01 (0.02)	0.02 (0.05)	0.01 (0.02)
***Amaranthus* spp**.	0.00 (0.00)	0.00 (0.00)	0.02 (0.05)	0.05 (0.13)
***Chloris* spp**.	0.00 (0.00)	0.07 (0.05)	0.00 (0.00)	0.05 (0.05)
***Aristida* spp**.	0.00 (0.00)	0.24 (0.16)	0.00 (0.00)	0.20 (0.16)

### Comparison of diets with the soil seed bank

**Fig 1:**
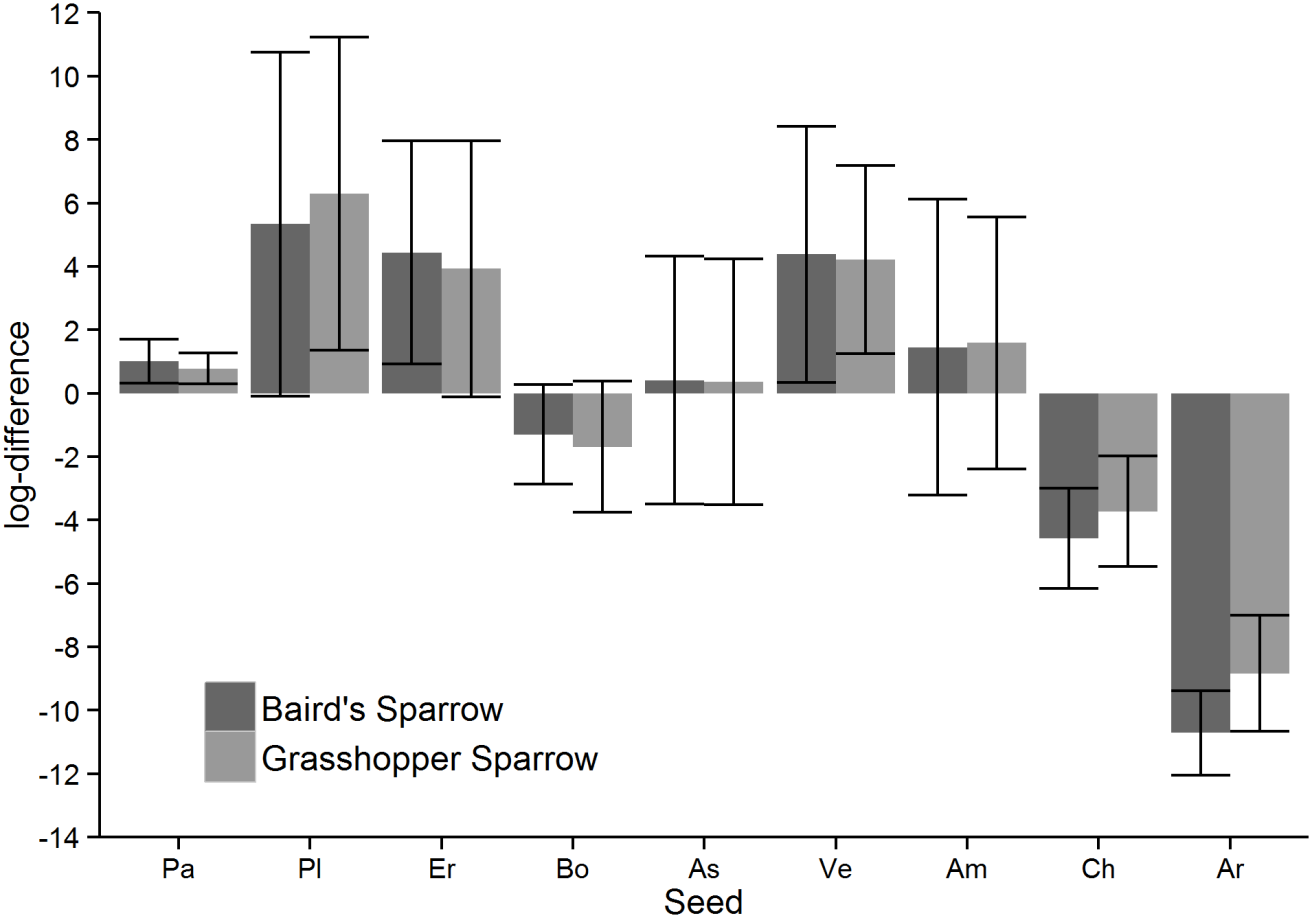
Log-ratios between seeds in diet and soil samples. A positive value means that the proportion of seeds in the diet is higher than in the soil, a negative value means that the proportion in the soil is higher than in the diet. Bars represent 95% confidence intervals. (Pa = Panicoideae, Pl = *Pleuraphis* spp., Er = Eragrostideae, Bo = *Bouteloua* spp., As = Asteraceae, Ve = *Verbena* spp., Am = *Amaranthus* spp., Ch = *Chloris* spp., Ar = *Aristida* spp.).

A comparison of the proportion of seeds in the soil samples with the proportion of seeds in diet using a Dirichlet regression showed that birds did not consume seeds according to their abundance, with the exception of *Amaranthus* (*B* = 0.25, *z* = 0.73, *df* = 18, *p* = 0.468) and Asteraceae (*B* = 0.31, *z* = 0.94, *df* = 18, *p* = 0.347; Fig 1). Panicoideae, *Pleuraphis*, Eragrostideae and *Verbena* were consumed more than would be expected based on their availability in the soil seed bank (*B* = 1.18, *z* = 4.23, *df* = 18, *p* < 0.001; *B* = 1.85, *z* = 5.70, *df* = 18, *p* < 0.001; *B* = 1.73, *z* = 5.38, *df* = 18, *p* < 0.001; *B* = 0.70, *z* = 2.08, *df* = 18, *p* = 0.038, respectively; Fig 1). *Bouteloua*, *Chloris*, and *Aristida* were consumed less than expected based on their availability (*B* = -0.60, *z* = -1.96, *df* = 18, *p* = 0.050; *B* = -0.99, *z* = -2.97, *df* = 18, *p* = 0.003; *B* = -2.53, *z* = -7.90 *df* = 18, *p* < 0.001, respectively; Fig 1). Diet composition did not differ significantly between bird species (*F* = 6.04, *df* = 1, 9, *p* = 0.306; Fig 1). Site and precipitation significantly affected the log-ratios between diet and soil composition (*F* = 14.40, *df* = 12, 27, p < 0.001; *F* = 6.73, *df* = 4, 9, *p* = 0.041) and there was suggestive but inconclusive evidence that sampling period influenced the log-ratios (*F* = 4.51, *df* = 4, 18, *p* = 0.077).

## Discussion

The stomach samples of Baird’s and Grasshopper sparrows overwintering in the Chihuahuan Desert of northern Mexico contained a large variety of seeds. However, the most consumed seeds belonged to a limited number of taxonomic groups. Preferred seeds (seeds that were consumed more than expected based on their availability in the soil seed bank) were not always the most consumed seeds; seeds from the genus *Bouteloua* were the second most consumed seeds on average and in some cases even the most consumed seeds, but they were not selected more than expected based on availability. Panicoideae were both common and preferred in the diet across all sites and sampling periods. Depending on their presence, birds also selected seeds from *Pleuraphis*, Eragrostideae and *Verbena*. *Amaranthus* and Asteraceae were consumed when present, but *Aristida* and *Chloris*, two common seeds in the soil seed bank, were avoided.

*Bouteloua* seeds were one of the most abundant seeds in the soil seed bank (Table 4). Seed-eating birds have been found to prefer seeds that they can handle most efficiently as to maximize energy intake over time [18,19,40,56]. It is possible that Baird’s and Grasshopper sparrows are able to increase energy intake over time by consuming more of the larger Panicoideae seeds compared to the smaller *Bouteloua* seeds. The large amount of *Bouteloua* seeds in Baird’s and Grasshopper sparrow diets suggests, however, that these seeds are important for their survival during the winter. Furthermore, birds did select these wintering sites with an abundance of *Bouteloua* seeds. In this regard, Baird’s and Grasshopper sparrows have previously been associated with *Bouteloua* [34]. *Bouteloua* grasses are native to the Chihuahuan Desert grasslands and because they are perennial grasses of moderate to high forage quality, they generally indicate a healthy grassland [57]. Baird’s and Grasshopper sparrows require dense vegetation with tall grasses [5]. Therefore, the vegetation characteristics associated with *Bouteloua* could also be important for wintering site selection by these sparrows.

Perennial grass seeds formed a large part of Baird’s and Grasshopper sparrow diets in the present study, in contrast to studies of other sparrow diets where forb and annual grass seeds were most important [6,29]. Desmond et al. [6] recognize that the dominance of annual grasses and forbs in their results might be due to disturbance of their study sites. Here, the study sites all consisted of grasslands dominated by native grasses, mainly perennial *Bouteloua* spp. and annual as well as perennial *Aristida* spp. However, *Aristida* was hardly consumed. In this regard, Desmond et al. [6] found that *Aristida* seeds were only important in sparrow diets in late winter after seed abundance had declined substantially. We only investigated diets in early and mid-winter and it is possible that Baird’s and Grasshopper sparrows include more *Aristida* in their diet towards the end of the winter. It has been suggested that sparrows would prefer forb over grass seeds because they are unarmored [29], although Marone et al. [16] found that sparrows of the Monte Desert in Argentina preferred grass over forb seeds. *Aristida* seeds have especially large awns which could explain why they are avoided. Although forbs did not form a large part of Baird’s and Grasshopper sparrow diets here, *Verbena* and *Amaranthus* were consumed when available.

Baird’s and Grasshopper sparrows were selective in their diet, showing preferences for some seeds and avoidance of others, but they did not differ in their preferences. Previously, we found that Grasshopper Sparrows are able to exploit larger seeds than Baird’s Sparrows, although there was a considerable overlap in preferences for seed species [35]. Grasshopper Sparrows have slightly larger bills than Baird’s Sparrows for which it may be expected that they can profitably consume larger or harder seeds [58,59,60]. However, the variability of seed size in the field may be small overall, limiting the potential for seed size partitioning between Baird’s and Grasshopper sparrows [20].

Seed availability was higher in 2013 for all sites, whereas precipitation was always higher in some sites compared to others (Table 2), irrespective of sampling period. This may explain the lack of a correlation between precipitation and seed availability, because both sites with higher and lower precipitation had more seeds in the last sampling period. Previous studies found a reduction of seeds in the soil seed bank from mid-winter to late winter [6,16,29]. Here seed abundance did not differ within a single season. However, we measured seed abundance in early winter (November) and mid-winter (January), in contrast to the other studies that compared mid-winter (January) to late winter (March).

Finally, it should be noted that there are some limitations to the use of DNA barcoding for the assessment of diets. First, the assumption that the proportion of sequence reads is equivalent to the actual proportion of a seed biomass in the diet may not be correct. Bias may occur during the different processing steps of the samples, especially PCR amplifications, because a small difference in amplification efficiency between two different seed species may result in a large difference in the amount of sequence copies after several PCR cycles [42]. Another source of error is variability between the seeds themselves in characteristics such as gene copy number or differences in the state of digestion [42], although the latter will also affect morphological seed identification. Nevertheless, comparisons of sequence-based data with traditional identification methods suggest that the proportion of sequence copies is a reasonable reflection of the actual proportion of a food item in the diet [48,61]. A second limitation is the resolution of the genetic barcode. Here we grouped species in higher taxonomic levels because the results on species level were not reliable. This is a common problem in diet studies using a DNA barcoding approach, e.g. [62]. Related to this is the fact that identification of food items is limited by the species available in the reference databank. In our case, the NCBI database did not contain all the species encountered in the study sites, which also made it necessary to group species into higher taxonomic levels. Comparing the food items in the diet to what is available, as we did with the soil seed bank, indicates what species are available for birds to select from and in what proportions. Large discrepancies between consumption and availability would indicate error. Therefore, the information on the soil seed bank composition and vegetation characteristics partly helped us to overcome the limitations mentioned above. However, we used different methods for identifying seeds in the stomach and crop contents compared to seeds in the soil seed bank, which could also produce bias. Ideally, we would have sequenced (part of) the soil samples to compare the different identification techniques, and we recommend doing this in future studies.

## Conclusion

Our results indicate which seeds are important for the winter diet of Baird’s and Grasshopper sparrows, despite the limitations mentioned above. This novel information provides a means to adequately assess habitat quality for wintering populations in Chihuahuan Desert grasslands. Although the diets consisted of a large variety of seeds, only a limited number of seeds was dominant. These seeds belonged to Panicoideae and *Bouteloua* spp. across sites and sampling periods, and *Pleuraphis* spp., Eragrostideae, Asteraceae, *Verbena* spp. and *Amaranthus* spp., depending on their availability. The dominance of Panicoideae and *Bouteloua* in the diets indicates the importance of perennial grasslands in the Chihuahuan Desert (usually dominated by species in these taxa) for the abundance and survival of overwintering Baird’s and Grasshopper sparrows, not only for shelter but also for food supply. We recommend promoting grassland management practices that maintain and restore native grassland species composition, for example by reducing grazing pressure during the growing season. Further research is necessary to determine exactly how management practices such as grazing intensity and timing affect seed production of preferred and dominant seeds in the diet, and how this affects Baird’s and Grasshopper sparrow wintering site selection and survival. It would furthermore be interesting to study the diet composition of these two birds in sites where Panicoideae are relatively absent and in sites with a higher degree of disturbance to gain more information on dietary flexibility in compromised situations. Finally, it is recommended that future molecular diet studies look for other DNA barcode regions such that seeds can be identified at a lower taxonomic level.

## Supporting information

S1 Appendix Botanical composition of the study sites compared to the soil seed bank. (XLSX)

S2 Appendix Datafile for statistical analysis. (XLSX)

S3 Appendix DNA barcoding results. (XLSX)
