# Supplementary material for: Molecular analysis of stomach contents reveals important grass seeds in the winter diet of Baird's and Grasshopper sparrows, two declining grassland bird species
Source: PLoS One. 2017 Dec 20;12(12):e0189695. doi: 10.1371/journal.pone.0189695 (PMC5738127; doi:10.1371/journal.pone.0189695)
Supplement: S1 Appendix — (XLSX) [file pone.0189695.s001.xlsx]

# S1 Appendix. Botanical composition of the study sites compared with the soil seed bank.

For seeds in the soil seed bank that were not encountered in the vegetation transects only those that had an abundance of 5% or more are reported.

**S1.1 Table. Botanical composition (percentage of total seed biomass) and soil seed bank at Santa Teresa, Durango, Mexico.**

| **Species** | **Dominance (%)** | **Soil Nov 2012 (%)** | **Soil Jan 2013 (%)** | **Soil Jan 2014 (%)** |
| --- | --- | --- | --- | --- |
| *Aristida* spp. (annual) | 3.8 | 6.22 | 7.29 | 16.55 |
| *Aristida* spp. (perennial) | 0.67 |  |  |  |
| Asteraceae | 0.56 | 20.51 | 27.49 | 46.68 |
| *Baccharis pteronioides* | 0.22 | 0 | 0 | 0 |
| *Botriochloa barbinodis* | 0.11 | 0 | 0 | 0 |
| *Bouteloua curtipendula* | 4.26 | 0 | 0 | 0.98 |
| *B. gracilis* | 34.19 | 13.36 | 17.23 | 3.09 |
| *B. hirsuta* | 0.11 | 0 | 0 | 0 |
| *Brickellia spinulosa* | 2.24 | 0 | 0 | 0 |
| *Buddleia scordioides* | 0.11 | 0 | 0 | 0 |
| *Chloris virgata* | 0.56 | 10.93 | 10.64 | 8.51 |
| *Condalia ericoides* | 1.01 | 0 | 0 | 0 |
| *Dichondria argentea* | 1.01 | 0 | 0 | 0 |
| *Dyschoriste schiedeana* | 0.34 | 0 | 0 | 0 |
| *Dyssodia papposa* | 0.11 | 0.91 | 0.03 | 0 |
| *Enneapogon desvauxii* | 7.62 | 6.71 | 9.42 | 0.22 |
| *Ephedra trifurca* | 0.45 | 0 | 0 | 0 |
| *Eragrostis cilianensis* | 0 | 0.14 | 0.76 | 6.78 |
| *Eupatorium odoratum* | 0.11 | 0 | 0 | 0 |
| *Eysenhardtia spinosa* | 1.01 | 0 | 0 | 0 |
| *E. texana* | 0.11 | 0 | 0 | 0 |
| *Juniperus monosperma* | 3.25 | 0 | 0 | 0 |
| *Mimosa biuncifera* | 0.11 | 0 | 0 | 0 |
| *Muhlenbergia phleoides* | 0.11 | 0 | 0 | 0.01 |
| *M. rigida* | 0.22 | 0 | 0.08 | 0 |
| *Panicum hallii* | 2.13 | 3.43 | 1.88 | 0.80 |
| *P. obtusum* | 0.11 | 0.19 | 0 | 0 |
| *Pectis papossa* | 2.45 | 0 | 0 | 0 |
| *Pleuraphis mutica* | 31.61 | 2.79 | 3.30 | 0.39 |
| *Prosopis glandulosa* | 0.11 | 0 | 0 | 0 |
| *Rhus microphya* | 0.45 | 0 | 0 | 0 |
| *Triquilia canescens* | 0.11 | 0 | 0 | 0 |
| *Zinnia grandiflora* | 0.11 | 0 | 0 | 0 |
| Unidentified 1 | 0.11 |  |  |  |
| Unidentified 2 | 0.11 |  |  |  |
| Unidentified 3 | 0.11 |  |  |  |

**S1.2 Table. Botanical composition (percentage of total seed biomass) and soil seed bank at Teseachi, Chihuahua, Mexico.**

| **Species** | **Dominance (%)** | **Soil Nov 2012 (%)** | **Soil Jan 2013 (%)** | **Soil Jan 2014 (%)** |
| --- | --- | --- | --- | --- |
| *Aristida* spp. | 8.04 | 7.47 | 5.79 | 52.97 |
| *Botriochloa barbinoides* | 0.10 | 0.44 | 0.08 | 0.12 |
| *Bouteloua curtipendula* | 0.19 | 0 | 0 | 0 |
| *B. gracilis* | 75.48 | 3.44 | 2.23 | 1.29 |
| *B. hirsuta* | 4.26 | 4.14 | 3.97 | 3.44 |
| *Chloris virgata* | 0 | 3.05 | 6.86 | 7.25 |
| *Commelina dianthifolia* | 0.10 | 0 | 0 | 0 |
| *Cyperus* spp. | 1.07 | 0 | 0 | 0 |
| *Elyonurus barbiculmis* | 0.10 | 0 | 0 | 0 |
| *Eragrostis* spp. | 0 | 2.67 | 7.56 | 10.87 |
| *Eriochloa* spp. | 0 | 6.37 | 19.77 | 5.27 |
| *Hymenoxys odorata* | 0 | 34.62 | 1.13 | 0 |
| *Lycurus phleoides* | 1.94 | 10.28 | 3.68 | 2.98 |
| *Muhlenbergia minutissima* | 0.97 | 0 | 0 | 0 |
| *M. rigida* | 3.78 | 0 | 0 | 0 |
| *Muhlenbergia* spp. | 0.19 | 0.84 | 0.24 | 0.02 |
| *Panicum bulbosum* | 0 | 0.46 | 1.00 | 6.60 |
| *Plantago patagonica* | 0.29 | 0 | 0 | 0 |
| *Polygonum aveniculare* | 0 | 8.07 | 16.41 | 1.39 |
| *Schizachyrium cirratum* | 2.81 | 0 | 0 | 0 |
| *Trachypogon secundus* | 0.10 | 0 | 0 | 0 |
| *Verbena neomexicana* | 0.39 | 3.24 | 9.37 | 0.65 |
| Unidentified | 0.20 |  |  |  |

**S1.3 Table. Botanial compostion (percentage of total seed biomass) and soil seed bank at El Uno – Centro, Chihuahua, Mexico.**

| **Species** | **Dominance (%)** | **Soil Nov 2012 (%)** | **Soil Jan 2013 (%)** | **Soil Jan 2014 (%)** |
| --- | --- | --- | --- | --- |
| *Acacia angustissima* | 0.21 | 0 | 0 | 0 |
| *Aristida adscencionis* | 4.83 | 4.40 | 12.66 | 3.78 |
| *A. orcutiana* | 1.05 | 0 | 0 | 0 |
| *Aristida* spp. (annual) | 8.19 | 0 | 0 | 0 |
| *Aristida* spp. (perennial) | 22.27 | 0 | 0 | 0 |
| *Atriplex tuberculata* | 0 | 70.48 | 46.86 | 1.30 |
| *Botriochloa barbinoides* | 1.26 | 0.44 | 0.98 | 2.18 |
| *Bouteloua barbata* | 0.63 | 1.07 | 0.01 | 3.55 |
| *B. eriopoda* | 24.37 | 0.22 | 2.07 | 0.63 |
| *B. gracilis* | 6.09 | 1.06 | 1.13 | 0 |
| *B. hirsuta* | 13.45 | 2.18 | 2.97 | 0.48 |
| *Ephedra trifurca* | 11.55 | 0 | 0 | 0 |
| *Eragrostis cilianensis* | 0 | 0 | 0 | 6.54 |
| *Evolvulus alsinoides* | 0.21 | 0 | 0 | 0 |
| *Haplopappus gracilis* | 0 | 4.02 | 6.93 | 2.15 |
| *Hofmansegia glauca* | 0.63 | 0 | 0 | 0 |
| *Machaeranthera pinnatifida* | 0.42 | 1.63 | 5.19 | 0.02 |
| *Mollugo verticillata* | 0 | 0 | 0 | 66.74 |
| *Panicum bulbosum* | 2.10 | 0.38 | 2.33 | 0 |
| *P. hirsutum* | 0.21 | 0.34 | 0.17 | 2.48 |
| *Pleuraphis mutica* | 0.21 | 0.22 | 0.08 | 0.54 |
| *Prosopis glandulosa* | 1.05 | 0 | 0 | 0 |
| *Salsola kali* | 0.42 | 0.57 | 2.12 | 2.04 |
| *Sida procumbens* | 0.21 | 0.01 | 0 | 0 |
| *Solanum elaeagnifolium* | 0.21 | 0 | 0 | 0 |
| *Zinnia acerosa* | 0.42 | 0 | 0 | 0 |

**S1.4 Table. Botanical composition (percentage of total seed biomass) and soil seed bank at El Uno – Los Ratones, Chihuahua, Mexico.**

| **Species** | **Dominance (%)** | **Soil Jan 2014 (%)** |
| --- | --- | --- |
| *Acacia angustissima* | 0.33 | 0 |
| *Amaranthus spp.* | 0.33 | 0 |
| *A. palmeri* | 0.50 | 35.20 |
| *Apodanthera undulata* | 0.33 | 0 |
| *Aristida adscensionis* | 40.13 | 4.79 |
| *A. longiseta* | 0.33 | 0 |
| *A. orcuttiana* | 0.84 | 0 |
| *Aristida* spp. (perennial) | 4.52 | 0 |
| *Botriochloa barbinodis* | 0.17 | 0.05 |
| *Bouteloua aristidoides* | 1.34 | 2.84 |
| *B. barbata* | 0.17 | 3.05 |
| *B. eriopoda* | 5.85 | 3.41 |
| *B. gracilis* | 9.03 | 0.02 |
| *B. hirsuta* | 0.33 | 0 |
| *Chloris virgata* | 0.17 | 0.53 |
| *Croton pottsii* | 0.33 | 0 |
| *Enneapogon desvauxii* | 0.17 | 0 |
| *Ephedra trifurca* | 0.17 | 0 |
| *Eragrostis cilianensis* | 0 | 12.11 |
| *E. superba* | 0 | 5.33 |
| *Evolvulus alsinoides* | 0.17 | 0 |
| *E. nuttallianus* | 0.33 | 0 |
| *Gutierrezia sarothrae* | 0.17 | 0 |
| *Pleuraphis mutica* | 24.08 | 0 |
| *Krameria grayi* | 0.17 | 0 |
| *Leguminosa* | 0.17 |  |
| *Machaeranthera pinnatifida* | 0.17 | 0 |
| *Mimosa biuncifera* | 0.17 | 0 |
| *Mollugo verticillata* | 0 | 8.09 |
| *Panicum obtusum* | 1.17 | 0.50 |
| *Panicum* spp. (annual) | 0.50 | 5.43 |
| *Prosopis glandulosa* | 3.51 | 0 |
| *Salsola iberica* | 2.17 | 0.31 |
| *Scleropogon brevifolius* | 0.67 | 0 |
| *Sida procumbens* | 0.33 | 0 |
| *Solanum elaeagnifolium* | 0.50 | 0 |
| *Sporobolus* spp. | 0.50 | 0 |
| Unidentified | 0.17 |  |
