# Supplementary material for: Molecular analysis of stomach contents reveals important grass seeds in the winter diet of Baird's and Grasshopper sparrows, two declining grassland bird species
Source: PLoS One. 2017 Dec 20;12(12):e0189695. doi: 10.1371/journal.pone.0189695 (PMC5738127; doi:10.1371/journal.pone.0189695)
Supplement: S3 Appendix — (XLSX) [file pone.0189695.s003.xlsx]

# S3 Appendix. DNA barcoding results.

**S3.1 Table. DNA barcoding results for pool 1: Baird’s Sparrow (*Ammodramus bairdii*), Santa Teresa, Durango, Mexico, November 2012.**

| **Genus** | **No. of reads** | **% of total reads** | **% of mapped reads** |
| --- | --- | --- | --- |
| *Botriochloa* | 488682 | 13.83 | 15.13 |
| *Panicum* | 430718 | 12.19 | 13.33 |
| *Setaria* | 314655 | 8.90 | 9.74 |
| *Hackelochloa* | 311347 | 8.81 | 9.64 |
| *Eriochloa* | 281978 | 7.98 | 8.73 |
| *Pleuraphis* | 225759 | 6.39 | 6.99 |
| *Bouteloua* | 187583 | 5.31 | 5.81 |
| *Muhlenbergia* | 179295 | 5.07 | 5.55 |
| *Lycurus* | 178832 | 5.06 | 5.54 |
| *Hypochaeris* | 171408 | 4.85 | 5.31 |
| *Verbena* | 65023 | 1.84 | 2.01 |
| *Verbesina* | 57449 | 1.63 | 1.78 |
| *Parthenium* | 57446 | 1.63 | 1.78 |
| *Helenium* | 57359 | 1.62 | 1.78 |
| *Amaranthus* | 53962 | 1.53 | 1.67 |
| *Eragrostis* | 48994 | 1.39 | 1.52 |
| *Enneapogon* | 44747 | 1.27 | 1.39 |
| *Bromus* | 24745 | 0.70 | 0.77 |
| *Machaeranthera* | 11174 | 0.32 | 0.35 |
| *Bidens* | 8331 | 0.24 | 0.26 |
| *Senecio* | 7679 | 0.22 | 0.24 |
| *Euphorbia* | 6581 | 0.19 | 0.20 |
| *Megathyrsus* | 4575 | 0.13 | 0.14 |
| *Gaura* | 4118 | 0.12 | 0.13 |
| *Chloris* | 1702 | 0.05 | 0.05 |
| *Datura* | 1486 | 0.04 | 0.05 |
| *Achillea* | 1123 | 0.03 | 0.03 |
| *Mollugo* | 957 | 0.03 | 0.03 |
| *Chenopodium* | 789 | 0.02 | 0.02 |
| *Descurainia* | 452 | 0.01 | 0.01 |
| *Portulaca* | 436 | 0.01 | 0.01 |
| *Solanum* | 143 |  |  |
| *Dyssodia* | 143 |  |  |
| *Elymus* | 138 |  |  |
| *Allium* | 80 |  |  |
| *Salsola* | 71 |  |  |
| *Brassica* | 47 |  |  |
| *Lamium* | 29 |  |  |
| *Aristida* | 25 |  |  |
| *Cucurbita* | 17 |  |  |
| *Schkuria* | 11 |  |  |
| *Plantago* | 6 |  |  |
| *Thlaspi* | 6 |  |  |
| *Sporobolus* | 5 |  |  |
| *Polygonum* | 2 |  |  |
| *Digitaria* | 2 |  |  |
| Read sum mapped | 3230110 |  |  |
| Read sum total | 3534032 |  |  |

**S3.2 Table. DNA barcoding results for pool 2: Grasshopper Sparrow (*Ammodramus savannarum*), Santa Teresa, Durango, Mexico November 2012.**

| **Genus** | **No. of reads** | **% of total reads** | **% of mapped reads** |
| --- | --- | --- | --- |
| *Bouteloua* | 101068 | 31.93 | 35.01 |
| *Hypochaeris* | 54087 | 17.09 | 18.73 |
| *Pleuraphis* | 23336 | 7.37 | 8.08 |
| *Parthenium* | 18175 | 5.74 | 6.30 |
| *Verbesina* | 18065 | 5.71 | 6.26 |
| *Helenium* | 18058 | 5.71 | 6.25 |
| *Panicum* | 11191 | 3.54 | 3.88 |
| *Euphorbia* | 10828 | 3.42 | 3.75 |
| *Eriochloa* | 10794 | 3.41 | 3.74 |
| *Setaria* | 4985 | 1.58 | 1.73 |
| *Hackelochloa* | 4952 | 1.56 | 1.72 |
| *Lycurus* | 4094 | 1.29 | 1.42 |
| *Muhlenbergia* | 4010 | 1.27 | 1.39 |
| *Senecio* | 1511 | 0.48 | 0.52 |
| *Botriochloa* | 1255 | 0.40 | 0.43 |
| *Bidens* | 510 | 0.16 | 0.18 |
| *Achillea* | 458 | 0.14 | 0.16 |
| *Eragrostis* | 329 | 0.10 | 0.11 |
| *Dyssodia* | 233 | 0.07 | 0.08 |
| *Solanum* | 216 | 0.07 | 0.07 |
| *Bromus* | 192 | 0.06 | 0.07 |
| *Chloris* | 109 | 0.03 | 0.04 |
| *Enneapogon* | 96 | 0.03 | 0.03 |
| *Megathyrsus* | 93 | 0.03 | 0.03 |
| *Datura* | 28 | 0.01 | 0.01 |
| *Verbena* | 19 | 0.01 | 0.01 |
| *Elymus* | 7 |  |  |
| *Machaeranthera* | 5 |  |  |
| *Schkuria* | 2 |  |  |
| *Allium* | 2 |  |  |
| *Aristida* | 1 |  |  |
| *Hymenoxys* | 1 |  |  |
| *Portulaca* | 1 |  |  |
| Read sum mapped | 288711 |  |  |
| Read sum total | 316490 |  |  |

**S3.3 Table. DNA barcoding results for pool 3: Baird’s Sparrow (*Ammodramus bairdii*), Teseachi,** **Chihuahua, Mexico, November 2012.**

| **Genus** | **No. of reads** | **% of total reads** | **% of mapped reads** |
| --- | --- | --- | --- |
| *Bouteloua* | 88757 | 29.16 | 31.92 |
| *Panicum* | 34588 | 11.36 | 12.44 |
| *Eriochloa* | 26921 | 8.85 | 9.68 |
| *Setaria* | 26517 | 8.71 | 9.54 |
| *Pleuraphis* | 16704 | 5.49 | 6.01 |
| *Muhlenbergia* | 15756 | 5.18 | 5.67 |
| *Lycurus* | 15599 | 5.13 | 5.61 |
| *Eragrostis* | 1206 | 0.40 | 0.43 |
| *Bromus* | 581 | 0.19 | 0.21 |
| *Megathyrsus* | 152 | 0.05 | 0.05 |
| *Chloris* | 141 | 0.05 | 0.05 |
| *Hypochaeris* | 135 | 0.04 | 0.05 |
| *Verbena* | 124 | 0.04 | 0.04 |
| *Euphorbia* | 115 | 0.04 | 0.04 |
| *Enneapogon* | 105 | 0.03 | 0.04 |
| *Parthenium* | 43 | 0.01 | 0.02 |
| *Helenium* | 43 | 0.01 | 0.02 |
| *Verbesina* | 37 | 0.01 | 0.01 |
| *Bidens* | 17 | 0.01 | 0.01 |
| *Brassica* | 15 |  | 0.01 |
| *Datura* | 10 |  |  |
| *Senecio* | 8 |  |  |
| *Machaeranthera* | 2 |  |  |
| *Allium* | 2 |  |  |
| *Cucurbita* | 1 |  |  |
| *Digitaria* | 1 |  |  |
| Read sum mapped | 278092 |  |  |
| Read sum total | 304364 |  |  |

**S3.4 Table. DNA barcoding results for pool 4: Grasshopper Sparrow (*Ammodramus savannarum*), Teseachi, Chihuahua, Mexico, November 2012.**

| **Genus** | **No. of reads** | **% of total reads** | **% of mapped reads** |
| --- | --- | --- | --- |
| *Bouteloua* | 19144 | 19.09 | 22.50 |
| *Eriochloa* | 16980 | 16.93 | 19.96 |
| *Panicum* | 11628 | 11.60 | 13.67 |
| *Setaria* | 8768 | 8.74 | 10.31 |
| *Hackelochloa* | 8188 | 8.17 | 9.63 |
| *Pleuraphis* | 4582 | 4.57 | 5.39 |
| *Botriochloa* | 4155 | 4.14 | 4.88 |
| *Muhlenbergia* | 3604 | 3.59 | 4.24 |
| *Lycurus* | 3487 | 3.48 | 4.10 |
| *Hypochaeris* | 1289 | 1.29 | 1.52 |
| *Eragrostis* | 532 | 0.53 | 0.63 |
| *Euphorbia* | 414 | 0.41 | 0.49 |
| *Verbesina* | 403 | 0.40 | 0.47 |
| *Parthenium* | 389 | 0.39 | 0.46 |
| *Helenium* | 384 | 0.38 | 0.45 |
| *Bromus* | 269 | 0.27 | 0.32 |
| *Megathyrsus* | 230 | 0.23 | 0.27 |
| *Enneapogon* | 205 | 0.20 | 0.24 |
| *Verbena* | 144 | 0.14 | 0.17 |
| *Senecio* | 99 | 0.10 | 0.12 |
| *Chloris* | 35 | 0.03 | 0.04 |
| *Bidens* | 24 | 0.02 | 0.03 |
| *Datura* | 23 | 0.02 | 0.03 |
| *Thlaspi* | 20 | 0.02 | 0.02 |
| *Brassica* | 18 | 0.02 | 0.02 |
| *Machaeranthera* | 12 | 0.01 | 0.01 |
| *Allium* | 10 | 0.01 | 0.01 |
| *Elymus* | 8 | 0.01 | 0.01 |
| *Cucurbita* | 7 | 0.01 | 0.01 |
| *Achillea* | 6 | 0.01 | 0.01 |
| *Dyssodia* | 3 |  |  |
| *Amaranthus* | 2 |  |  |
| *Aristida* | 2 |  |  |
| *Descurainia* | 1 |  |  |
| *Portulaca* | 1 |  |  |
| Read sum mapped | 85066 |  |  |
| Read sum total | 100273 |  |  |

**S3.5 Table. DNA barcoding results for pool 5: Baird’s Sparrow (*Ammodramus bairdii*), El Uno - Centro, Chihuahua, Mexico, November 2012.**

| **Genus** | **No. of reads** | **% of total reads** | **% of mapped reads** |
| --- | --- | --- | --- |
| *Panicum* | 66942 | 25.46 | 27.81 |
| *Botriochloa* | 51076 | 19.43 | 21.22 |
| *Hackelochloa* | 50494 | 19.21 | 20.98 |
| *Setaria* | 50329 | 19.14 | 20.91 |
| *Bouteloua* | 17034 | 6.48 | 7.08 |
| *Machaeranthera* | 1589 | 0.60 | 0.66 |
| *Eriochloa* | 878 | 0.33 | 0.36 |
| *Pleuraphis* | 399 | 0.15 | 0.17 |
| *Muhlenbergia* | 290 | 0.11 | 0.12 |
| *Lycurus* | 257 | 0.10 | 0.11 |
| *Eragrostis* | 251 | 0.10 | 0.10 |
| *Chloris* | 187 | 0.07 | 0.08 |
| *Hypochaeris* | 168 | 0.06 | 0.07 |
| *Enneapogon* | 131 | 0.05 | 0.05 |
| *Bromus* | 106 | 0.04 | 0.04 |
| *Gaura* | 105 | 0.04 | 0.04 |
| *Parthenium* | 68 | 0.03 | 0.03 |
| *Verbesina* | 53 | 0.02 | 0.02 |
| *Elymus* | 51 | 0.02 | 0.02 |
| *Helenium* | 50 | 0.02 | 0.02 |
| *Chenopodium* | 50 | 0.02 | 0.02 |
| *Megathyrsus* | 45 | 0.02 | 0.02 |
| *Amaranthus* | 39 | 0.01 | 0.02 |
| *Solanum* | 26 | 0.01 | 0.01 |
| *Aristida* | 17 | 0.01 | 0.01 |
| *Senecio* | 12 |  |  |
| *Bidens* | 11 |  |  |
| *Euphorbia* | 9 |  |  |
| *Verbena* | 9 |  |  |
| *Datura* | 5 |  |  |
| *Lamium* | 2 |  |  |
| *Cucurbita* | 2 |  |  |
| *Dyssodia* | 1 |  |  |
| *Achillea* | 1 |  |  |
| Read sum mapped | 240687 |  |  |
| Read sum total | 262910 |  |  |

**S3.6 Table. DNA barcoding results for pool 6: Grasshopper Sparrow (*Ammodramus savannarum*), El Uno - Centro, Chihuahua, Mexico, November 2012.**

| **Genus** | **No. of reads** | **% of total reads** | **% of mapped reads** |
| --- | --- | --- | --- |
| *Panicum* | 54241 | 24.32 | 27.34 |
| *Hackelochloa* | 40866 | 18.32 | 20.60 |
| *Setaria* | 40754 | 18.27 | 20.54 |
| *Botriochloa* | 19016 | 8.53 | 9.58 |
| *Pleuraphis* | 12883 | 5.78 | 6.49 |
| *Lycurus* | 12732 | 5.71 | 6.42 |
| *Muhlenbergia* | 12674 | 5.68 | 6.39 |
| *Machaeranthera* | 2066 | 0.93 | 1.04 |
| *Eriochloa* | 1026 | 0.46 | 0.52 |
| *Chloris* | 278 | 0.12 | 0.14 |
| *Bouteloua* | 266 | 0.12 | 0.13 |
| *Chenopodium* | 238 | 0.11 | 0.12 |
| *Hypochaeris* | 218 | 0.10 | 0.11 |
| *Elymus* | 203 | 0.09 | 0.10 |
| *Eragrostis* | 192 | 0.09 | 0.10 |
| *Euphorbia* | 150 | 0.07 | 0.08 |
| *Enneapogon* | 112 | 0.05 | 0.06 |
| *Bromus* | 87 | 0.04 | 0.04 |
| *Allium* | 78 | 0.03 | 0.04 |
| *Helenium* | 74 | 0.03 | 0.04 |
| *Parthenium* | 68 | 0.03 | 0.03 |
| *Verbesina* | 62 | 0.03 | 0.03 |
| *Megathyrsus* | 22 | 0.01 | 0.01 |
| *Salsola* | 20 | 0.01 | 0.01 |
| *Descurainia* | 17 | 0.01 | 0.01 |
| *Aristida* | 11 |  | 0.01 |
| *Senecio* | 10 |  | 0.01 |
| *Bidens* | 7 |  |  |
| *Amaranthus* | 7 |  |  |
| *Verbena* | 6 |  |  |
| *Datura* | 4 |  |  |
| *Thlaspi* | 3 |  |  |
| *Brassica* | 1 |  |  |
| *Mollugo* | 1 |  |  |
| *Sporobolus* | 1 |  |  |
| Read sum mapped | 198394 |  |  |
| Read sum total | 223029 |  |  |

**S3.7 Table. DNA barcoding results for pool 7: Baird’s Sparrow (*Ammodramus bairdii*), Santa Teresa, Durango, Mexico, January 2013.**

| **Genus** | **No. of reads** | **% of total reads** | **% of mapped reads** |
| --- | --- | --- | --- |
| *Pleuraphis* | 118152 | 30.30 | 32.35 |
| *Botriochloa* | 49292 | 12.64 | 13.50 |
| *Panicum* | 36830 | 9.44 | 10.09 |
| *Eriochloa* | 35525 | 9.11 | 9.73 |
| *Setaria* | 26350 | 6.76 | 7.22 |
| *Hackelochloa* | 26271 | 6.74 | 7.19 |
| *Bouteloua* | 22735 | 5.83 | 6.23 |
| *Enneapogon* | 10957 | 2.81 | 3.00 |
| *Lycurus* | 10693 | 2.74 | 2.93 |
| *Muhlenbergia* | 10544 | 2.70 | 2.89 |
| *Hypochaeris* | 6139 | 1.57 | 1.68 |
| *Helenium* | 2139 | 0.55 | 0.59 |
| *Parthenium* | 2108 | 0.54 | 0.58 |
| *Verbesina* | 2033 | 0.52 | 0.56 |
| *Eragrostis* | 1237 | 0.32 | 0.34 |
| *Euphorbia* | 925 | 0.24 | 0.25 |
| *Megathyrsus* | 875 | 0.22 | 0.24 |
| *Bromus* | 566 | 0.15 | 0.15 |
| *Verbena* | 552 | 0.14 | 0.15 |
| *Chloris* | 289 | 0.07 | 0.08 |
| *Gaura* | 247 | 0.06 | 0.07 |
| *Elymus* | 201 | 0.05 | 0.06 |
| *Senecio* | 146 | 0.04 | 0.04 |
| *Bidens* | 76 | 0.02 | 0.02 |
| *Descurainia* | 61 | 0.02 | 0.02 |
| *Allium* | 60 | 0.02 | 0.02 |
| *Thlaspi* | 55 | 0.01 | 0.02 |
| *Salsola* | 43 | 0.01 | 0.01 |
| *Brassica* | 32 | 0.01 | 0.01 |
| *Datura* | 16 |  |  |
| *Dyssodia* | 12 |  |  |
| *Achillea* | 9 |  |  |
| *Schkuria* | 5 |  |  |
| *Solanum* | 4 |  |  |
| *Amaranthus* | 3 |  |  |
| *Chenopodium* | 1 |  |  |
| *Machaeranthera* | 1 |  |  |
| *Cucurbita* | 1 |  |  |
| Read sum mapped | 365185 |  |  |
| Read sum total | 389963 |  |  |

**S3.8 Table. DNA barcoding results for pool 8: Grasshopper Sparrow (*Ammodramus savannarum*), Santa Teresa, Durango, Mexico, January 2013.**

| **Genus** | **No. of reads** | **% of total reads** | **% of mapped reads** |
| --- | --- | --- | --- |
| *Bouteloua* | 146057 | 34.69 | 37.15 |
| *Eriochloa* | 47938 | 11.39 | 12.19 |
| *Pleuraphis* | 37609 | 8.93 | 9.57 |
| *Panicum* | 34454 | 8.18 | 8.76 |
| *Setaria* | 24728 | 5.87 | 6.29 |
| *Hackelochloa* | 24538 | 5.83 | 6.24 |
| *Botriochloa* | 16175 | 3.84 | 4.11 |
| *Muhlenbergia* | 15439 | 3.67 | 3.93 |
| *Lycurus* | 15177 | 3.60 | 3.86 |
| *Datura* | 14133 | 3.36 | 3.59 |
| *Enneapogon* | 9487 | 2.25 | 2.41 |
| *Hypochaeris* | 1946 | 0.46 | 0.49 |
| *Euphorbia* | 898 | 0.21 | 0.23 |
| *Megathyrsus* | 780 | 0.19 | 0.20 |
| *Helenium* | 696 | 0.17 | 0.18 |
| *Parthenium* | 633 | 0.15 | 0.16 |
| *Verbesina* | 622 | 0.15 | 0.16 |
| *Eragrostis* | 588 | 0.14 | 0.15 |
| *Bromus* | 426 | 0.10 | 0.11 |
| *Verbena* | 354 | 0.08 | 0.09 |
| *Chloris* | 206 | 0.05 | 0.05 |
| *Senecio* | 95 | 0.02 | 0.02 |
| *Allium* | 41 | 0.01 | 0.01 |
| *Elymus* | 33 | 0.01 | 0.01 |
| *Machaeranthera* | 22 | 0.01 | 0.01 |
| *Gaura* | 20 |  | 0.01 |
| *Portulaca* | 18 |  |  |
| *Thlaspi* | 10 |  |  |
| *Bidens* | 9 |  |  |
| *Solanum* | 9 |  |  |
| *Achillea* | 2 |  |  |
| *Cucurbita* | 1 |  |  |
| *Aristida* | 1 |  |  |
| *Dyssodia* | 1 |  |  |
| Read sum mapped | 393146 |  |  |
| Read sum total | 421051 |  |  |

**S3.9 Table. DNA barcoding results for pool 9: Baird’s Sparrow (*Ammodramus bairdii*), Teseachi, Chihuahua, Mexico, January 2013.**

| **Genus** | **No. of reads** | **% of total reads** | **% of mapped reads** |
| --- | --- | --- | --- |
| *Verbena* | 94815 | 23.34 | 24.76 |
| *Panicum* | 52380 | 12.89 | 13.68 |
| *Hackelochloa* | 39036 | 9.61 | 10.20 |
| *Setaria* | 39010 | 9.60 | 10.19 |
| *Hackelochloa* | 39036 | 9.61 | 10.20 |
| *Hypochaeris* | 28984 | 7.13 | 7.57 |
| *Eriochloa* | 26556 | 6.54 | 6.94 |
| *Pleuraphis* | 15804 | 3.89 | 4.13 |
| *Muhlenbergia* | 14255 | 3.51 | 3.72 |
| *Lycurus* | 14202 | 3.50 | 3.71 |
| *Bouteloua* | 13949 | 3.43 | 3.64 |
| *Botriochloa* | 10347 | 2.55 | 2.70 |
| *Helenium* | 9705 | 2.39 | 2.53 |
| *Verbesina* | 9657 | 2.38 | 2.52 |
| *Parthenium* | 9503 | 2.34 | 2.48 |
| *Senecio* | 1502 | 0.37 | 0.39 |
| *Bidens* | 1383 | 0.34 | 0.36 |
| *Eragrostis* | 806 | 0.20 | 0.21 |
| *Bromus* | 423 | 0.10 | 0.11 |
| *Megathyrsus* | 145 | 0.04 | 0.04 |
| *Enneapogon* | 123 | 0.03 | 0.03 |
| *Euphorbia* | 83 | 0.02 | 0.02 |
| *Achillea* | 66 | 0.02 | 0.02 |
| *Allium* | 59 | 0.01 | 0.02 |
| *Chloris* | 22 | 0.01 | 0.01 |
| *Dyssodia* | 19 |  |  |
| *Datura* | 19 |  |  |
| *Solanum* | 9 |  |  |
| *Thlaspi* | 4 |  |  |
| *Brassica* | 4 |  |  |
| *Elymus* | 2 |  |  |
| *Machaeranthera* | 2 |  |  |
| *Descurainia* | 1 |  |  |
| *Schkuria* | 1 |  |  |
| *Hymenoxys* | 1 |  |  |
| Read sum mapped | 382877 |  |  |
| Read sum total | 406244 |  |  |

**S3.10 Table. DNA barcoding results for pool 10: Grasshopper Sparrow (*Ammodramus savannarum*), Teseachi, Chihuahua, Mexico, January 2013.**

| **Genus** | **No. of reads** | **% of total reads** | **% of mapped reads** |
| --- | --- | --- | --- |
| *Eriochloa* | 115819 | 27.02 | 29.04 |
| *Verbena* | 53122 | 12.39 | 13.32 |
| *Hypochaeris* | 52650 | 12.28 | 13.20 |
| *Panicum* | 20516 | 4.79 | 5.14 |
| *Helenium* | 17641 | 4.12 | 4.42 |
| *Verbesina* | 17476 | 4.08 | 4.38 |
| *Parthenium* | 17468 | 4.07 | 4.38 |
| *Bouteloua* | 14029 | 3.27 | 3.52 |
| *Botriochloa* | 13848 | 3.23 | 3.47 |
| *Eragrostis* | 13117 | 3.06 | 3.29 |
| *Hackelochloa* | 12899 | 3.01 | 3.23 |
| *Setaria* | 12781 | 2.98 | 3.20 |
| *Pleuraphis* | 10804 | 2.52 | 2.71 |
| *Lycurus* | 7556 | 1.76 | 1.89 |
| *Muhlenbergia* | 7431 | 1.73 | 1.86 |
| *Bromus* | 6668 | 1.56 | 1.67 |
| *Senecio* | 2895 | 0.68 | 0.73 |
| *Megathyrsus* | 1533 | 0.36 | 0.38 |
| *Bidens* | 167 | 0.04 | 0.04 |
| *Achillea* | 153 | 0.04 | 0.04 |
| *Enneapogon* | 93 | 0.02 | 0.02 |
| *Chloris* | 88 | 0.02 | 0.02 |
| *Dyssodia* | 27 | 0.01 | 0.01 |
| *Euphorbia* | 16 |  |  |
| *Elymus* | 9 |  |  |
| *Allium* | 8 |  |  |
| *Datura* | 7 |  |  |
| *Schkuria* | 4 |  |  |
| *Brassica* | 4 |  |  |
| *Cucurbita* | 4 |  |  |
| *Machaeranthera* | 3 |  |  |
| *Aristida* | 1 |  |  |
| Read sum mapped | 398837 |  |  |
| Read sum total | 428691 |  |  |

**S3.11 Table. DNA barcoding results for pool 11: Baird’s Sparrow (*Ammodramus bairdii*), El Uno - Centro, Chihuahua, Mexico, January 2013.**

| **Genus** | **No. of reads** | **% of total reads** | **% of mapped reads** |
| --- | --- | --- | --- |
| *Botriochloa* | 147307 | 34.65 | 37.44 |
| *Panicum* | 65826 | 15.48 | 16.73 |
| *Setaria* | 49437 | 11.63 | 12.57 |
| *Hackelochloa* | 49354 | 11.61 | 12.54 |
| *Pleuraphis* | 21919 | 5.16 | 5.57 |
| *Muhlenbergia* | 21520 | 5.06 | 5.47 |
| *Lycurus* | 21488 | 5.05 | 5.46 |
| *Machaeranthera* | 6866 | 1.61 | 1.75 |
| *Eriochloa* | 4354 | 1.02 | 1.11 |
| *Datura* | 1419 | 0.33 | 0.36 |
| *Chenopodium* | 771 | 0.18 | 0.20 |
| *Hypochaeris* | 652 | 0.15 | 0.17 |
| *Bouteloua* | 461 | 0.11 | 0.12 |
| *Eragrostis* | 302 | 0.07 | 0.08 |
| *Parthenium* | 235 | 0.06 | 0.06 |
| *Verbesina* | 234 | 0.06 | 0.06 |
| *Helenium* | 234 | 0.06 | 0.06 |
| *Euphorbia* | 168 | 0.04 | 0.04 |
| *Bromus* | 160 | 0.04 | 0.04 |
| *Chloris* | 149 | 0.04 | 0.04 |
| *Verbena* | 138 | 0.03 | 0.04 |
| *Megathyrsus* | 106 | 0.02 | 0.03 |
| *Enneapogon* | 101 | 0.02 | 0.03 |
| *Gaura* | 54 | 0.01 | 0.01 |
| *Descurainia* | 45 | 0.01 | 0.01 |
| *Senecio* | 25 | 0.01 | 0.01 |
| *Bidens* | 23 | 0.01 | 0.01 |
| *Brassica* | 21 |  | 0.01 |
| *Allium* | 14 |  |  |
| *Elymus* | 12 |  |  |
| *Aristida* | 10 |  |  |
| *Thlaspi* | 6 |  |  |
| *Salsola* | 4 |  |  |
| *Amaranthus* | 4 |  |  |
| *Achillea* | 4 |  |  |
| *Solanum* | 3 |  |  |
| *Sporobolus* | 2 |  |  |
| *Cucurbita* | 1 |  |  |
| *Mollugo* | 1 |  |  |
| *Dyssodia* | 1 |  |  |
| Read sum mapped | 393431 |  |  |
| Read sum total | 425142 |  |  |

**S3.12 Table. DNA barcoding results for pool 12: Grasshopper Sparrow (*Ammodramus savannarum*), El Uno - Centro, Chihuahua, Mexico, January 2013.**

| **Genus** | **No. of reads** | **% of total reads** | **% of mapped reads** |
| --- | --- | --- | --- |
| *Botriochloa* | 84870 | 21.95 | 23.86 |
| *Panicum* | 45242 | 11.70 | 12.72 |
| *Pleuraphis* | 39062 | 10.10 | 10.98 |
| *Muhlenbergia* | 38422 | 9.94 | 10.80 |
| *Lycurus* | 38120 | 9.86 | 10.72 |
| *Setaria* | 33689 | 8.71 | 9.47 |
| *Amaranthus* | 3943 | 1.02 | 1.11 |
| *Hypochaeris* | 2995 | 0.77 | 0.84 |
| *Bouteloua* | 2703 | 0.70 | 0.76 |
| *Euphorbia* | 1103 | 0.29 | 0.31 |
| *Verbesina* | 1057 | 0.27 | 0.30 |
| *Helenium* | 1055 | 0.27 | 0.30 |
| *Parthenium* | 1052 | 0.27 | 0.30 |
| *Eragrostis* | 842 | 0.22 | 0.24 |
| *Megathyrsus* | 541 | 0.14 | 0.15 |
| *Enneapogon* | 462 | 0.12 | 0.13 |
| *Bromus* | 391 | 0.10 | 0.11 |
| *Machaeranthera* | 339 | 0.09 | 0.10 |
| *Verbena* | 291 | 0.08 | 0.08 |
| *Chloris* | 187 | 0.05 | 0.05 |
| *Senecio* | 156 | 0.04 | 0.04 |
| *Salsola* | 49 | 0.01 | 0.01 |
| *Datura* | 25 | 0.01 | 0.01 |
| *Elymus* | 24 | 0.01 | 0.01 |
| *Lamium* | 22 | 0.01 | 0.01 |
| *Brassica* | 19 |  | 0.01 |
| *Bidens* | 9 |  |  |
| *Cucurbita* | 9 |  |  |
| *Aristida* | 8 |  |  |
| *Chenopodium* | 6 |  |  |
| *Achillea* | 5 |  |  |
| *Dyssodia* | 4 |  |  |
| *Allium* | 4 |  |  |
| *Solanum* | 2 |  |  |
| *Sporobolus* | 1 |  |  |
| Read sum mapped | 355715 |  |  |
| Read sum total | 386686 |  |  |

**S3.13 Table. DNA barcoding results for pool 13: Baird’s Sparrow (*Ammodramus bairdii*), Santa Teresa, Durango, Mexico, January 2014.**

| **Genus** | **No. of reads** | **% of total reads** | **% of mapped reads** |
| --- | --- | --- | --- |
| *Hypochaeris* | 81537 | 18.21 | 19.78 |
| *Pleuraphis* | 59620 | 13.31 | 14.46 |
| *Muhlenbergia* | 47288 | 10.56 | 11.47 |
| *Lycurus* | 47186 | 10.54 | 11.45 |
| *Enneapogon* | 28866 | 6.45 | 7.00 |
| *Verbesina* | 27163 | 6.07 | 6.59 |
| *Helenium* | 27143 | 6.06 | 6.58 |
| *Parthenium* | 27022 | 6.03 | 6.56 |
| *Eragrostis* | 17581 | 3.93 | 4.27 |
| *Bromus* | 8848 | 1.98 | 2.15 |
| *Bidens* | 7205 | 1.61 | 1.75 |
| *Eriochloa* | 6461 | 1.44 | 1.57 |
| *Panicum* | 5460 | 1.22 | 1.32 |
| *Bouteloua* | 4846 | 1.08 | 1.18 |
| *Gaura* | 4053 | 0.90 | 0.98 |
| *Senecio* | 3635 | 0.81 | 0.88 |
| *Hackelochloa* | 2455 | 0.55 | 0.60 |
| *Setaria* | 2402 | 0.54 | 0.58 |
| *Machaeranthera* | 1766 | 0.39 | 0.43 |
| *Achillea* | 731 | 0.16 | 0.18 |
| *Chloris* | 596 | 0.13 | 0.14 |
| *Botriochloa* | 163 | 0.04 | 0.04 |
| *Megathyrsus* | 61 | 0.01 | 0.01 |
| *Euphorbia* | 47 | 0.01 | 0.01 |
| *Dyssodia* | 43 | 0.01 | 0.01 |
| *Amaranthus* | 12 |  |  |
| *Verbena* | 4 |  |  |
| *Lamium* | 4 |  |  |
| *Allium* | 3 |  |  |
| *Schkuria* | 2 |  |  |
| *Aristida* | 2 |  |  |
| *Elymus* | 1 |  |  |
| *Chenopodium* | 1 |  |  |
| *Brassica* | 1 |  |  |
| *Portulaca* | 1 |  |  |
| Read sum mapped | 412209 |  |  |
| Read sum total | 447858 |  |  |

**S3.14 Table. DNA barcoding results for pool 14: Baird’s Sparrow (*Ammodramus bairdii*), Teseachi, Chihuahua, Mexico, January 2014.**

| **Genus** | **No. of reads** | **% of total reads** | **% of mapped reads** |
| --- | --- | --- | --- |
| *Botriochloa* | 120833 | 28.21 | 32.73 |
| *Bouteloua* | 55802 | 13.03 | 15.12 |
| *Pleuraphis* | 26306 | 6.14 | 7.13 |
| *Panicum* | 26049 | 6.08 | 7.06 |
| *Muhlenbergia* | 25258 | 5.90 | 6.84 |
| *Lycurus* | 25084 | 5.86 | 6.80 |
| *Eriochloa* | 22749 | 5.31 | 6.16 |
| *Hackelochloa* | 18616 | 4.35 | 5.04 |
| *Setaria* | 18599 | 4.34 | 5.04 |
| *Eragrostis* | 13463 | 3.14 | 3.65 |
| *Verbena* | 7564 | 1.77 | 2.05 |
| *Bromus* | 6857 | 1.60 | 1.86 |
| *Megathyrsus* | 712 | 0.17 | 0.19 |
| *Hypochaeris* | 536 | 0.13 | 0.15 |
| *Parthenium* | 191 | 0.04 | 0.05 |
| *Verbesina* | 188 | 0.04 | 0.05 |
| *Helenium* | 185 | 0.04 | 0.05 |
| *Chloris* | 46 | 0.01 | 0.01 |
| *Senecio* | 31 | 0.01 | 0.01 |
| *Bidens* | 24 | 0.01 | 0.01 |
| *Enneapogon* | 22 | 0.01 | 0.01 |
| *Amaranthus* | 8 |  |  |
| *Achillea* | 8 |  |  |
| *Datura* | 3 |  |  |
| *Gaura* | 3 |  |  |
| *Digitaria* | 2 |  |  |
| *Salsola* | 1 |  |  |
| *Polygonum* | 1 |  |  |
| *Euphorbia* | 1 |  |  |
| *Cucurbita* | 1 |  |  |
| *Dyssodia* | 1 |  |  |
| *Elymus* | 1 |  |  |
| Read sum mapped | 369145 |  |  |
| Read sum total | 428304 |  |  |

**S3.15 Table. DNA barcoding results for pool 15: Grasshopper Sparrow (*Ammodramus savannarum*), Teseachi, Chihuahua, Mexico, January 2014.**

| **Genus** | **No. of reads** | **% of total reads** | **% of mapped reads** |
| --- | --- | --- | --- |
| *Botriochloa* | 97495 | 22.76 | 24.90 |
| *Eriochloa* | 56701 | 13.24 | 14.48 |
| *Panicum* | 53979 | 12.60 | 13.78 |
| *Bouteloua* | 42466 | 9.91 | 10.84 |
| *Setaria* | 38314 | 8.94 | 9.78 |
| *Hackelochloa* | 38260 | 8.93 | 9.77 |
| *Pleuraphis* | 20345 | 4.75 | 5.20 |
| *Muhlenbergia* | 18675 | 4.36 | 4.77 |
| *Lycurus* | 18490 | 4.32 | 4.72 |
| *Verbena* | 2597 | 0.61 | 0.66 |
| *Megathyrsus* | 1236 | 0.29 | 0.32 |
| *Machaeranthera* | 957 | 0.22 | 0.24 |
| *Eragrostis* | 811 | 0.19 | 0.21 |
| *Bromus* | 438 | 0.10 | 0.11 |
| *Hypochaeris* | 242 | 0.06 | 0.06 |
| *Verbesina* | 87 | 0.02 | 0.02 |
| *Helenium* | 75 | 0.02 | 0.02 |
| *Parthenium* | 74 | 0.02 | 0.02 |
| *Euphorbia* | 74 | 0.02 | 0.02 |
| *Elymus* | 74 | 0.02 | 0.02 |
| *Chloris* | 67 | 0.02 | 0.02 |
| *Enneapogon* | 54 | 0.01 | 0.01 |
| *Dyssodia* | 47 | 0.01 | 0.01 |
| *Bidens* | 14 |  |  |
| *Senecio* | 9 |  |  |
| *Amaranthus* | 9 |  |  |
| *Datura* | 6 |  |  |
| *Achillea* | 2 |  |  |
| *Gaura* | 2 |  |  |
| *Aristida* | 2 |  |  |
| *Sporobolus* | 1 |  |  |
| *Brassica* | 1 |  |  |
| Read sum mapped | 391604 |  |  |
| Read sum total | 428372 |  |  |

**S3.16 Table. DNA barcoding results for pool 16: Grasshopper Sparrow (*Ammodramus savannarum*), El Uno – Centro, Chihuahua, Mexico, January 2014.**

| **Genus** | **No. of reads** | **% of total reads** | **% of mapped reads** |
| --- | --- | --- | --- |
| *Panicum* | 134300 | 31.44 | 34.21 |
| *Setaria* | 102103 | 23.90 | 26.01 |
| *Hackelochloa* | 100613 | 23.55 | 25.63 |
| *Botriochloa* | 19814 | 4.64 | 5.05 |
| *Pleuraphis* | 13320 | 3.12 | 3.39 |
| *Lycurus* | 7594 | 1.78 | 1.93 |
| *Muhlenbergia* | 7377 | 1.73 | 1.88 |
| *Eriochloa* | 1808 | 0.42 | 0.46 |
| *Eragrostis* | 1370 | 0.32 | 0.35 |
| *Mollugo* | 903 | 0.21 | 0.23 |
| *Bromus* | 725 | 0.17 | 0.18 |
| *Machaeranthera* | 663 | 0.16 | 0.17 |
| *Descurainia* | 407 | 0.10 | 0.10 |
| *Portulaca* | 362 | 0.08 | 0.09 |
| *Chloris* | 316 | 0.07 | 0.08 |
| *Hypochaeris* | 169 | 0.04 | 0.04 |
| *Bouteloua* | 136 | 0.03 | 0.03 |
| *Bidens* | 73 | 0.02 | 0.02 |
| *Helenium* | 72 | 0.02 | 0.02 |
| *Enneapogon* | 69 | 0.02 | 0.02 |
| *Verbesina* | 67 | 0.02 | 0.02 |
| *Megathyrsus* | 66 | 0.02 | 0.02 |
| *Parthenium* | 62 | 0.01 | 0.02 |
| *Verbena* | 39 | 0.01 | 0.01 |
| *Euphorbia* | 20 |  | 0.01 |
| *Salsola* | 16 |  |  |
| *Elymus* | 14 |  |  |
| *Senecio* | 14 |  |  |
| *Datura* | 11 |  |  |
| *Amaranthus* | 9 |  |  |
| *Chenopodium* | 9 |  |  |
| *Plantago* | 4 |  |  |
| *Gaura* | 2 |  |  |
| *Aristida* | 2 |  |  |
| *Lamium* | 1 |  |  |
| *Brassica* | 1 |  |  |
| Read sum mapped | 392531 |  |  |
| Read sum total | 427170 |  |  |

**S3.17 Table. DNA barcoding results for pool 17: Grasshopper Sparrow (*Ammodramus savannarum*), El Uno – Los Ratones, Chihuahua, Mexico, January 2014.**

| **Genus** | **No. of reads** | **% of total reads** | **% of mapped reads** |
| --- | --- | --- | --- |
| *Panicum* | 70873 | 20.01 | 21.39 |
| *Setaria* | 53636 | 15.15 | 16.19 |
| *Hackelochloa* | 53351 | 15.07 | 16.10 |
| *Amaranthus* | 49975 | 14.11 | 15.08 |
| *Eriochloa* | 47410 | 13.39 | 14.31 |
| *Bouteloua* | 20896 | 5.90 | 6.31 |
| *Pleuraphis* | 12332 | 3.48 | 3.72 |
| *Muhlenbergia* | 8121 | 2.29 | 2.45 |
| *Lycurus* | 8058 | 2.28 | 2.43 |
| *Euphorbia* | 2822 | 0.80 | 0.85 |
| *Botriochloa* | 1313 | 0.37 | 0.40 |
| *Eragrostis* | 856 | 0.24 | 0.26 |
| *Machaeranthera* | 571 | 0.16 | 0.17 |
| *Bromus* | 420 | 0.12 | 0.13 |
| *Megathyrsus* | 178 | 0.05 | 0.05 |
| *Chloris* | 121 | 0.03 | 0.04 |
| *Hypochaeris* | 94 | 0.03 | 0.03 |
| *Enneapogon* | 77 | 0.02 | 0.02 |
| *Mollugo* | 53 | 0.01 | 0.02 |
| *Verbesina* | 40 | 0.01 | 0.01 |
| *Parthenium* | 35 | 0.01 | 0.01 |
| *Helenium* | 27 | 0.01 | 0.01 |
| *Bidens* | 13 |  |  |
| *Verbena* | 9 |  |  |
| *Datura* | 9 |  |  |
| *Senecio* | 5 |  |  |
| *Achillea* | 4 |  |  |
| *Solanum* | 3 |  |  |
| *Chenopodium* | 2 |  |  |
| *Lamium* | 2 |  |  |
| *Plantago* | 2 |  |  |
| *Salsola* | 1 |  |  |
| *Sporobolus* | 1 |  |  |
| *Allium* | 1 |  |  |
| *Polygonum* | 1 |  |  |
| Read sum mapped | 331312 |  |  |
| Read sum total | 354111 |  |  |
